# The Impact of the German Strategy for Containment of Coronavirus SARS-CoV-2 on Training Characteristics, Physical Activity and Sleep of Highly Trained Kayakers and Canoeists: A Retrospective Observational Study

**DOI:** 10.3389/fspor.2020.579830

**Published:** 2020-10-15

**Authors:** Christoph Zinner, Manuel Matzka, Robert Leppich, Samuel Kounev, Hans-Christer Holmberg, Billy Sperlich

**Affiliations:** ^1^Department of Sport, University of Applied Sciences for Police and Administration of Hesse, Wiesbaden, Germany; ^2^Integrative and Experimental Training Science, Department of Sport Science, University of Würzburg, Würzburg, Germany; ^3^Software Engineering Group, Department of Computer Science, University of Würzburg, Würzburg, Germany; ^4^Department of Physiology and Pharmacology, Biomedicum C5, Karolinska Institutet, Stockholm, Sweden; ^5^Biomechanics Laboratory, Beijing Sport University, Beijing, China

**Keywords:** COVID-19, coronavirus, pandemic (COVID-19), remote monitoring, training intensity distribution, wearables, wearable technology

## Abstract

**Aim:** To characterize the impact of the German strategy for containment of Coronavirus SARS-CoV-2 (social distancing and lockdown) on the training, other habitual physical activity, and sleep in highly trained kayakers and canoeists.

**Method:** During the 4 weeks immediately prior to and following the beginning of the German government's strategy for containment of Coronavirus SARS-CoV-2 on March 23, 2020, 14 highly trained athletes (VO_2peak_: 3,162 ± 774 ml/min; 500-m best time: 117.9 ± 7.9 s) wore a multi-sensor smartwatch to allow continuous assessment of heart rate, physical activity, and sleep duration.

**Result:** In comparison to before lockdown, the overall weekly training time and the average length of each session of training during the lockdown decreased by 27.6% (*P* = 0.02; *d* = 0.91) and 15.4% (*P* = 0.36; *d* = 0.36), respectively. At the same time, the number of sessions involving specific (i.e., canoeing and kayaking) and non-specific (i.e., running, cycling) training, respectively, did not change (*P* = 0.36–0.37; *d* = 0.34–0.35). The number of sessions involving strength (+17.4%; *P* = 0.03; *d* = 0.89) or other types of training (+16.7%; *P* = 0.06; *d* = 0.75) increased during the lockdown with 2.8–17.5% more training time involving a heart rate <60%, 82–88, 89–93, or 94–100% of individual peak heart rate (HR_peak_) (*P* = 0.03–0.86; *d* = 0.07–1.38), and 4.3–18.7% less time with a heart rate of 60–72 or 73–83% HR_peak_ (*P* = < 0.001–0.0.26; *d* = 0.44–2.24). The daily duration of sleep was ~30 min (6.7%) longer during the lockdown (*P* < 0.001; *d* = 1.53) and the overall time spent lying down was 17% greater (*P* < 0.001; *d* = 2.26); whereas sitting time (−9.4%; *P* = 0.003; *d* = 1.23), the duration of light (15 min; −7.3%; *P* = 0.04; *d* = 0.83), and moderate (−18.6%; *P* = 0.01; *d* = 1.00) physical activity other than training (−9.4%; *P* = 0.22; *d* = 0.00) were all lower during lockdown.

**Conclusion:** The present data revealed that following the German lockdown for containment of the Coronavirus SARS-CoV-2, highly trained kayakers and canoeists spent less overall time training each week (−27.6%) with, on average, shorter training sessions (−15.1%) and less light-to-moderate physical activity outside of training. Moreover, they performed more strength training sessions per week, and all engaged in more training at intensities >82 and <60% of HR_peak_ and spent longer periods lying down and sleeping during the lockdown.

## Introduction

On January 30, 2020, the World Health Organization (WHO) declared the outbreak of COVID-19 (coronavirus disease) to be a public health emergency of international concern. Countermeasures to reduce the risk of infection include improved hygiene, social distancing, and forbidding large social gatherings such as sport events. Indeed, nearly all international and national athletic competitions, including the 2020 Olympic games, have been postponed.

More specifically, as of March 23, 2020, the official regulations for containment of Coronavirus SARS-CoV-2 in Germany included prohibition of group gatherings, including closing of training facilities (e.g., sports halls, gyms, swimming pools, etc.) for organized sports. This abrupt change has forced athletes and coaches to adopt different training strategies in preparation for competitions hoped to be held in late summer. At the same time, in contrast to other parts of the world (e.g., Spain and Italy), the German strategy allowed individuals to exercise without the direct (i.e., face-to-face) assistance of coaches and without using indoor training facilities.

As mentioned earlier (Sarto et al., 2020), when athletes, especially those who are welltrained, are confined to their homes, their individual beneficial cardiorespiratory and neuromuscular adaptations to various types and extents of physical activity are likely to be lost, at least to some extent (Mujika and Padilla, 2000). In elite athletes such adaptions, as well as offtraining behavior (in particular habitual physical activity) and sleep (Sperlich and Holmberg, 2017), are key determinants of performance. Most athletes who normally engage in frequent and intense training are likely to be forced to alter these determinants during the lockdown. The manner and degree to which these alterations take place are presently unknown.

Here, we retrospectively examine the effects of the present pandemic situation in Germany on the intensity and duration of training, other types of physical activity, sleep, and certain physiological characteristics related to the performance of elite kayakers. For this purpose, we compare data collected with sensors during 4 weeks prior to and 4 weeks during the lockdown.

## Methods

### Participants

The key anthropometric, physiological, and performance characteristics of the highly trained flatwater sprint kayakers (four men and eight women) and canoeists (two men) who participated are summarized in Table 1. To ensure anonymity, we present age groups and body mass index instead of the exact age, body mass, and height.

**Table 1:** The sex, age, body mass index, discipline (*C* = canoe, *K* = kayak), personal best times*, and peak oxygen uptake before lockdown (*best competition time from season 2019; n.d. = not determined).

**P-ID**	**Sex**	**Age group (yrs)**	**Body Mass Index**	**Discipline**	Personal best time* **(s)**			**% Of world best time**			**Peak oxygen**
			**(kg/m^**2**^)**								**uptake (ml/min)**
					**200 m**	**500 m**	**1,000 m**	**200 m**	**500 m**	**1,000 m**	**Before lockdown**
1	male	Under 18	24.3	C	<44.0	<117.9	<260.8	85.2	88.1	85.3	4,161
2	male	Under 18	23.5	C	<47.4	<124.5	<287.6	79.1	83.4	77.4	3,519
3	male	Under 18	24.1	K	<40.3	<106.8	<243.4	83.1	89.3	83.3	3,436
4	male	Under 18	22.5	K	n.d.	<108.9	<240.6	n.d.	87.5	84.2	4,326
5	male	Under 23	23.1	K	n.d.	<103.0	<221.5	n.d.	92.5	91.5	4,485
6	male	Under 18	22.8	K	<42.8	<116.0	<235.5	80.1	82.1	86.1	3,686
7	female	Under 18	23.6	K	<45.0	<122.5	n.d.	84.4	87.0	n.d.	2,402
8	female	Under 18	23.3	K	<48.8	<124.4	<270.3	77.8	85.7	84.6	2,410
9	female	Under 18	22.0	K	<45.4	<120.8	<264.6	83.7	88.2	86.5	2,481
10	female	Under 23	25.1	K	<43.2	<115.9	n.d.	88.0	92.0	n.d.	2,970
11	female	Under 18	20.4	K	<50.0	<131.4	<279.6	76.1	81.1	81.8	2,741
12	female	Under 18	20.3	K	<46.9	<122.5	n.d.	81.0	87.0	n.d.	2,190
13	female	Under 18	21.9	K	<46.6	<119.6	n.d.	81.6	89.1	n.d.	2,554
14	female	Under 18	24.0	K	<49.8	n.d.	n.d.	78.0	n.d.	n.d.	2,905
Mean ± SD		17.1 ± 1.9	22.9 ± 1.4		45.6 ± 2.9	117.9 ± 7.9	255.8 ± 22.0				3,162 ± 774

Six are presently members of the German Development Team and eight of the West-German Regional Team, and they all compete at least at the highest national level in Germany. All procedures were preapproved by the institute's ethics committee and conducted in accordance with the Declaration of Helsinki. In addition, after being informed in detail about the risks, benefits, and procedures of this study, all participants and their legal guardians provided their written consent for participation.

### Observation

During the entire 4 weeks prior to and after the announcement on March 23, 2020, of the strategy for containment of Coronavirus SARS-CoV-2 by the German government, all participants wore a multi-sensor smartwatch (Polar M430, Polar OY, Kempele, Finland) that collected data on their heart rate during each training session and other physical activities, as well as data on the duration of sleep and duration of sleep. Eight weeks before the lockdown, all of the athletes underwent routine diagnostic tests of performance, including incremental testing designed to determine peak oxygen uptake (VO_2peak_) and heart rate (HR_peak_). All testing on-water took place on official regatta courses in North-Rhine Westphalia, Germany, venues on which some of the international canoe sprint championships, as well as the World Cup series organized by the International Canoe Federation (ICF) were held.

### Training Modes and Their Documentation

All kayakers and canoeist recorded their type (e.g., kayaking, running, cycling, etc.), duration, distance, and intensity of training, as well as information on days off and illness or injury using their smartwatch. These diaries, which were immediately uploaded to the Polar Server, were checked by the coaches and cross-checked by the research team for plausibility. After completion of the study, all data were exported as.tcx files for analysis.

All training sessions were divided into four different modes, namely: (i) Kayaking/Canoeing in a kayak/canoe or on an ergometer; (ii) other kinds of *Endurance* training, such as running, cycling, swimming, etc.; (iii) *Strength* and resistance training either with machines or weights or against body mass; and (iv) Other: stretching, stability training, etc.

### The Distribution of Training Intensity

Training intensity was divided into five zones on the basis of heart rate (Seiler, 2010), which was monitored during each session of training by a chest belt that communicated with the athlete's smartwatch. This allowed calculation of the time spent in each intensity zone for subsequent analysis.

### Assessment of Physical Activity (PA) and Sleep Duration

Since extensive concomitant daily physical activity may improve responses to endurance training (Hautala et al., 2012), physical activity when the participants were not training was also monitored continuously by the multisensory device (Polar M430) worn on the wrist for the entire experimental period, being removed only for charging. The reliability of the Polar M430 under such free-living conditions has been validated and its suitability to report changes in physical activity over time (Henriksen et al., 2019). Comparison of the algorithm utilized to assess sleep time with polysomnography in adolescents revealed that the former underestimates slightly but less than many accelerometers designed for research purposes (Pesonen and Kuula, 2018). We employed established classifications (Tremblay et al., 2017) by dividing the data collected into sedentary [i.e., lying and sitting with <1.5 metabolic equivalent of task (MET)], light (1.5–3 MET), moderate (3–6 MET), or vigorous (>6 MET) energy expenditure (in min·d^−1^) utilizing the Polar Online Software.

### Evaluation of VO_2peak_ and Body Mass Index

The incremental test protocol involved 4 × 1,500 m trials on-water at 70, 80, and 90% of HR_peak_, as well as an all-out effort. All athletes were members of the Canoe Federation and therefore highly experienced in performing this type of protocol.

The heart rate utilized here was based on the HR_peak_ determined previously by the Western German Canoe Federation using this same protocol. The 30–45 s that elapsed between successive steps was required for sampling capillary blood from the earlobe. All participants received continuous visual feedback concerning their heart rate (averaged every second) from a monitor (Polar Wear Link System and Polar V800 Heart Rate Monitor, Polar Electro OY, Kempele, Finland) mounted directly in front of them on the boat or ergometer. At all points, the strokerate was self-selected.

Oxygen uptake was monitored continuously with an open-circuit breath-by-breath analyzer (MetaMax 3B, Cortex Biophysik, Leipzig, Germany), employing standard algorithms to compensate for the time delay between gas consumption and the signal. This analyzer was calibrated prior to each test with both 15.8% O_2_ and 5% CO_2_ in N_2_ (Praxair, Düsseldorf, Germany); that is, concentrations that cover the range of expected fractional concentrations of O_2_. The volume sensor was calibrated with a precision 3-L syringe (Cortex Biophysik, Leipzig, Germany). VO_2_ was averaged every 30 s during the test and the highest value considered to be VO_2peak_.

#### Statistical Analysis

The data used to calculate the MET values were processed with the Python analysis toolkit “pandas” (1.0.3) available for the Python programming language (3.8). In addition, utilizing the Statistica software package for Windows® (version 7.1, StatSoft Inc., Tulsa, OK, USA), Student's paired *t*-test was applied to identify potential differences between the sedentary time and light, moderate, and vigorous physical activity on weekdays and weekend with an alpha of *P* < 0.05 being considered statistically significant. Moreover, the effect size, Cohen's *d*, (Cohen, 1988) was calculated for all variables with the thresholds for small, moderate, and large effects set to 0.20, 0.50, and 0.80, respectively (Cohen, 1988). Medium or large effect sizes associated with insignificant *p*-values were considered to indicate tendencies.

## Results

### Training Sessions

The number of different training sessions (canoeing or kayaking, strength, endurance, other), the overall training time, the average duration of each session, and the days without training prior to and during the lockdown are summarized in Table 2.

**Table 2:** Numbers of training sessions, overall training time and duration of each session during the 4 weeks prior to and the initial 4 weeks of lockdown.

	**Week before lockdown**				**Week during lockdown**				**Mean**				
	**−4**	**−3**	**−2**	**−1**	**1**	**2**	**3**	**4**	**Before lockdown**	**During lockdown**	**%-Difference**	***P***	***d***
Overall training time (min)	858 ± 248	656 ± 448	1,511 ± 842	804 ± 476	855 ± 545	800 ± 393	567 ± 233	548 ± 485	957 ± 379	693 ± 158	−27.6	0.02	0.91
Duration of each session (min)	62 ± 24	61 ± 24	91 ± 74	78 ± 22	65 ± 21	76 ± 19	58 ± 9	52 ± 16	73 ± 40	62 ± 18	−15.1	0.36	0.36
**Number of training sessions**													
-specific endurance	5.9 ± 1.8	3.9 ± 1.7	5.9 ± 1.8	4.0 ± 2.2	5.4 ± 1.6	5.8 ± 1.6	4.9 ± 1.1	4.9 ± 2.5	4.9 ± 1.1	5.2 ± 0.5	+6.1	0.36	0.35
(canoeing and kayaking)													
-non-specific endurance	1.9 ± 1.0	1.6 ± 1.0	1.9 ± 1.0	2.5 ± 1.3	2.0 ± 1.4	1.9 ± 0.8	2.0 ± 1.0	1.8 ± 1.2	2.0 ± 0.4	1.9 ± 0.1	−0.05	0.37	0.34
(running, cycling etc.)													
-strength	2.4 ± 1.0	2.6 ± 1.4	2.2 ± 1.4	2.2 ± 1.3	3.6 ± 1.3	2.7 ± 1.4	2.4 ± 1.2	2.2 ± 1.6	2.3 ± 0.2	2.7 ± 0.6	+17.4	0.03	0.89
-other^*^	2.3 ± 1.5	1.5 ± 0.7	1.5 ± 0.9	2.2 ± 1.5	2.3 ± 1.8	2.0 ± 1.6	2.7 ± 2.0	1.7 ± 1.1	1.8 ± 0.4	2.1 ± 0.4	+16.7	0.06	0.75

* *Including stretching, basketball, mobility training, indoor sports, and other indoor activities*.

The overall training time per week and average duration of each session were 27.6% and 15.4% lower, respectively, during the lockdown. The number of sessions involving specific (i.e., canoeing or kayaking) and non-specific (i.e., running, cycling) training remained similar during both periods. The number of sessions involving strength or other types of training (e.g., stretching) was 17.4% and 16.7% higher, respectively, before the lockdown began.

### The Distribution of Training Intensity

**Figure 1:**
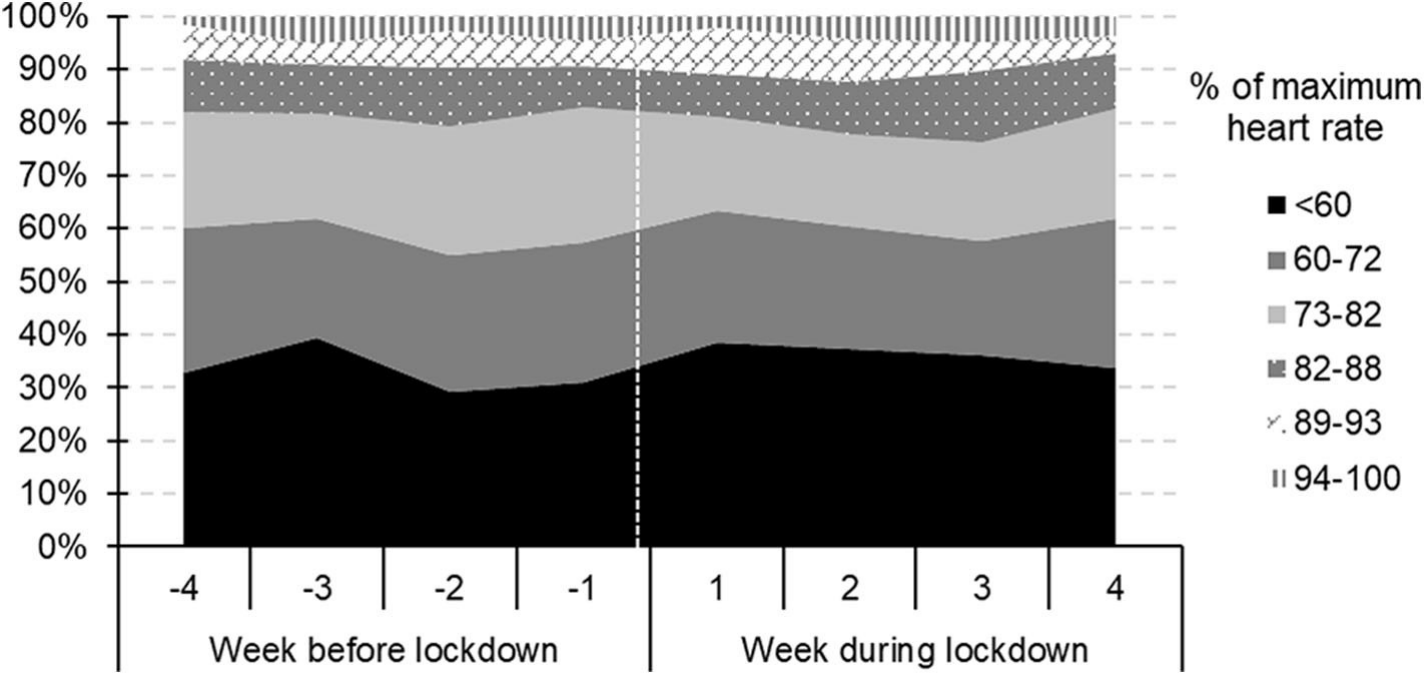
The proportions of the total training time each week spent in the different intensity zones during the 4-week periods prior to and at the beginning of the lockdown.

The fractions of the total training time spent in each intensity zone are summarized in Table 3 and illustrated in Figure 1.

**Table 3:** The mean percentage of the total training time each week spent in each intensity zone during the 4-weeks periods prior to and at the beginning of the lockdown.

	**% of peak heart rate**	**Week before lockdown**				**Week during lockdown**				**Mean**				
		**−4**	**−3**	**−2**	**−1**	**1**	**2**	**3**	**4**	**Before lockdown**	**During lockdown**	**%-Difference**	***P***	***d***
%-time	94–100	1.6 ± 1.4	5.2 ± 8.3	2.7 ± 3.3	4.8 ± 5.3	2.1 ± 2.4	4.4 ± 6.6	4.9 ± 4.8	3.7 ± 3.3	3.6 ± 1.7	3.7 ± 1.2	+2.8	0.86	0.07
in zone	89–93	6.7 ± 5.0	4.2 ± 5.8	7.0 ± 9.5	4.9 ± 8.0	9.2 ± 12.7	8.6 ± 7.4	5.6 ± 3.7	3.4 ± 2.8	5.7 ± 1.4	6.7 ± 2.7	+17.5	0.23	0.47
	82–88	10.2 ± 6.0	9.6 ± 7.6	11.3 ± 10.0	8.0 ± 5.6	8.2 ± 5.5	10.3 ± 7.9	13.7 ± 12.7	10.8 ± 8.5	9.8 ± 1.4	10.8 ± 2.3	+10.2	0.18	0.53
	73–82	22.6 ± 11.9	20.6 ± 17.0	24.8 ± 14.0	26.0 ± 16.6	18.2 ± 10.7	18.2 ± 11.5	19.0 ± 12.9	21.1 ± 11.7	23.5 ± 2.4	19.1 ± 1.4	−18.7	<0.001	2.24
	60–72	28.2 ± 13.8	23.1 ± 11.3	26.4 ± 13.0	27.2 ± 11.4	25.6 ± 15.5	24.2 ± 13.2	22.1 ± 11.7	28.7 ± 11.9	26.2 ± 2.2	25.1 ± 2.8	−4.2	0.26	0.44
	<60	34.0 ± 13.2	40.9 ± 18.2	29.8 ± 12.1	31.8 ± 18.9	39.7 ± 11.2	38.9 ± 14.1	36.9 ± 20.4	34.3 ± 15.1	34.1 ± 4.8	37.4 ± 2.4	+9.7	0.03	1.38

Prior to the lockdown 2.8–17.5% more training time involved heart rates <60, 82–88, 89–93, and 94–100% of individual HR_peak_, with 4.3–18.7% less training time involving heart rates of 60–72 and 73–83% of individual HR_peak._

### Sedentary Time, Time Spent Doing Other Physical Activities, and Duration of Sleep

**Figure 2:**
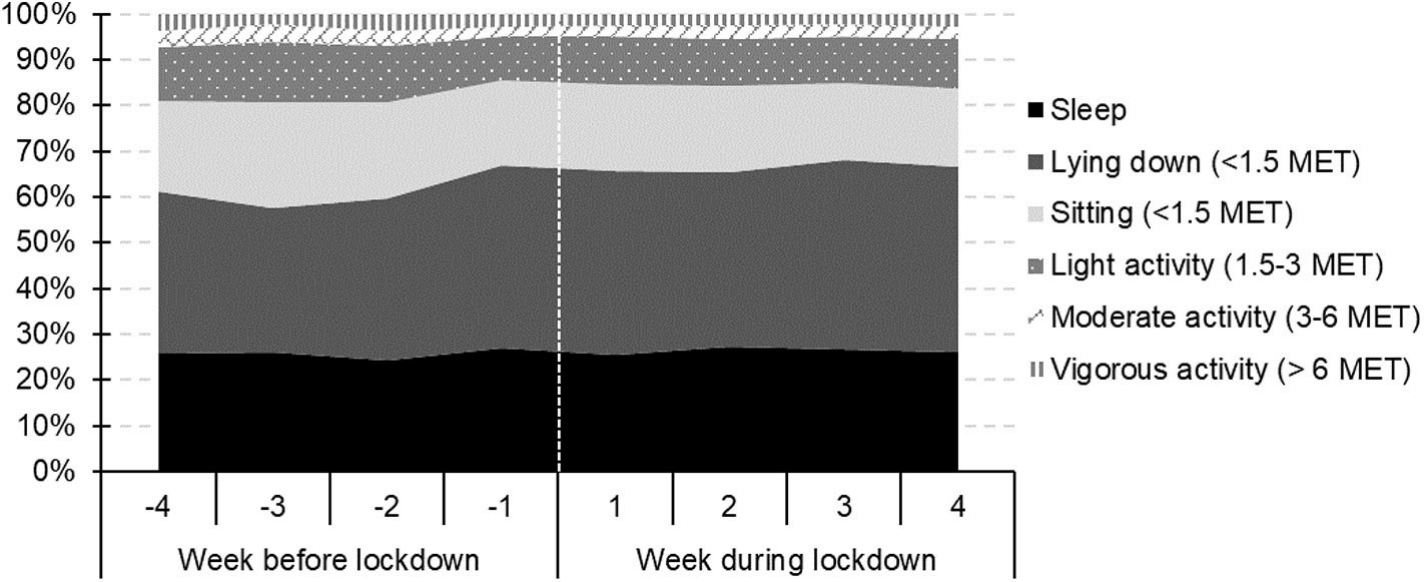
The proportional duration of sleep, lying down and sitting, and physical activities other than training during the 4-week periods immediately before and after the start of the lockdown (MET = metabolic equivalent of task).

Overall, the average daily duration of sleep was ~30 min (6.7%) longer during the lockdown (see Table 4 and Figure 2). The average time spent lying down was 106 min (17%) greater during the lockdown, while the time spent sitting was 34 min (9.4%) lower. In addition, the times spent performing light (15 min = 7.3%) and moderate (11 min = 18.6%) physical activity all became shorter once the lockdown was put in place.

**Table 4:** Mean duration (in minutes) of sleep, sedentary time and time spent doing other physical activities during the 4-week periods immediately before and after the start of the lockdown (MET = metabolic equivalent of task).

**Activity**	**Week before lockdown**				**Week during lockdown**				**Mean**				
	**−4**	**−3**	**−2**	**−1**	**1**	**2**	**3**	**4**	**Before lockdown**	**During lockdown**	**% Difference**	***P***	***d***
Sleep	460 ± 39	452 ± 60	419 ± 65	470 ± 82	464 ± 78	504 ± 47	481 ± 96	476 ± 41	451 ± 22	481 ± 17	+6.7	<0.001	1.53
Lying down (<1.5 MET)	632 ± 93	547 ± 105	613 ± 135	701 ± 160	725 ± 159	703 ± 144	752 ± 174	736 ± 197	623 ± 63	729 ± 21	+17.0	<0.001	2.26
Sitting (<1.5 MET)	351 ± 72	402 ± 88	366 ± 99	322 ± 126	342 ± 108	346 ± 129	305 ± 141	312 ± 121	360 ± 33	326 ± 21	−9.4	0.003	1.23
Light activity (1.5–3 MET)	208 ± 54	230 ± 70	213 ± 74	170 ± 66	191 ± 57	190 ± 60	184 ± 83	196 ± 80	205 ± 25	190 ± 5	−7.3	0.04	0.83
Moderate activity (3–6 MET)	67 ± 25	72 ± 27	57 ± 17	38 ± 24	43 ± 21	53 ± 22	48 ± 25	48 ± 26	59 ± 15	48 ± 4	−18.6	0.01	1.00
Vigorous activity (>6 MET)	66 ± 20	36 ± 24	65 ± 32	48 ± 29	48 ± 19	47 ± 11	42 ± 22	53 ± 36	53 ± 14	48 ± 5	−9.4	0.22	0.48

## Discussion

In light of the potential influence of the corona lockdown in Germany on the physical status of elite athletes, this observational study compared the training, other habitual physical activities, and sleep duration of well-trained and elite kayakers and canoeists during the 4-week periods immediately before and after this lockdown was put in place (Sarto et al., 2020). The major changes associated with the lockdown were as follows:

1. Overall training time per week and the average duration of each session decreased by 27.6% and 15.4%, respectively.
2. Our athletes performed 17.4% more strength training and 16.7% more sessions of other types of training each week.
3. There was no change in the number of specific and non-specific sessions of training, respectively.
4. The mean portion of the total training time spent at <60, 82–88, 89–93, or 94–100% of maximum heart rate was 2.8–17.5% greater and 4.3–18.7% lower at 60–72 or 73–83% of the individual HR_peak_, respectively.
5. On average, each athlete slept ~30 min (6.6%) longer.
6. On average, 17% more time was spent lying down, whereas the times spent sitting (−9.4%), or performing light (−7.3%) or moderate (−18.6%) physical activity other than training were all lower.

Typically, the long-term strategy used by athletes to prepare for competition involves periods of exercise (usually 2–4-week meso-cycles) varying in their amount, intensity, and frequency. Here, we analyzed two 4-weeks periods during the Spring preparation by kayakers and canoeists (usually lasting from the beginning of February to the end of April). Our findings indicate that the German strategy for the containment of the Coronavirus SARS-CoV-2 (which allowed individual activities outdoors) reduced the overall time during which elite kayakers trained each week by ~25%, with an average of 15.4% decrease in the duration of each training session.

Among our athletes, this reduction was probably related to changes in training due to the lockdown. The coaches immediately altered training schedules so that underage athletes did not train onwater alone, and all boat houses were closed in order to avoid group gatherings, which meant that some athletes had no access to their boats at the beginning of the lockdown. At this point in the season, a reduction in overall training time is unusual. Nonetheless, it is clear that our German kayakers and canoeists did not reduce their training stimulus significantly during the initial period of lockdown, as suspected earlier (Sarto et al., 2020), still averaging 693 ± 158 min of training per week. It would be interesting to compare this result to the impact of stricter containment of Coronavirus SARS-CoV-2 (e.g., remaining indoors with no specific endurance training) enforced by certain other countries on the training of elite athletes. In contrast, other countries such as Sweden enforced less strict measures to contain Coronavirus SARS-CoV-2 and we might hypothesize that in these countries the seasonal preparation (besides international traveling) was not heavily influenced.

Interestingly, the numbers of specific and non-specific training sessions before and following the lockdown were similar, indicating that our group of athletes made use of the opportunity to engage in specific endurance training. Overall, the kayakers and canoeists performed more sessions of strength training per week, although our approach does not allow characterization of the type of strength training they engaged in.

Our present investigation focused on the effects of the German lockdown only during the initial 4 weeks, that is for a relatively short period of time. It remains to be determined whether the impact on long-term preparation for national championships and/or qualification for international championships might differ. The observation that the kayakers and canoeists trained to a greater extent at intensities of >82 and <60% of HR_peak_ during the lockdown were related to the highly unusual situation; such changes and especially the reduction in training time were certainly not considered desirable by the coaches.

Adequate sleep exerts an important impact in connection with the optimization of both the physical and cognitive performance of elite athletes, as well as in reducing the risk of injury (Charest and Grandner, 2020). Our athletes slept ~30 min (6.6%) longer each day during the German containment (i.e., 8 h rather than 7.5 h). Previous reports indicate that Olympic athletes sleep less (6.5–6.8 h) than the 8 h recommended daily (Leeder et al., 2012; Lastella et al., 2015). There are a number of possible explanations as to why our athletes slept more during the lockdown: (i) they may have adopted less rigorous morning training schedules (Sargent et al., 2014; Gupta et al., 2017); (ii) the German containment strategy significantly reduced international travel obligations and opportunities; (iii) they engaged to a greater extent in home-schooling and -office; (iv) the stress and anxiety related to upcoming qualification events were no longer present (Erlacher et al., 2011; Juliff et al., 2015), or (v) some athletes may have developed some forms of depression due to social distancing and lack of prospects. Unfortunately, the monitoring methodology we employed does not allow assessment of the quality of sleep (sleep latency and efficiency).

Interestingly, the overall time spent lying down each day (including sleep) was 17% (+106 min) greater, while the sitting time decreased by 9.4% during the lockdown, indicating more time for passive recovery. The potential reasons for the longer time spent lying down are similar to those mentioned above for the longer sleeping time.

The finding that the extents of light (−7.3%), moderate (−18.6%), and vigorous (−9.4%) physical activity other than training were lower during the lockdown is not surprising, since all types of social activity (commuting, social interactions in larger groups, etc.) were limited drastically by the German strategy for containment of Coronavirus SARS-CoV-2. Overall, our group of athletes were more active than other groups in the population-based studies (Hagstromer et al., 2007, 2010; Hansen et al., 2012).

### Methodological Considerations

Some methodological considerations/limitations associated with the current observational investigation need to be acknowledged: (i) since the overall observation period involved only 4 weeks before and 4 weeks during the initial phase of lockdown, our findings cannot easily be generalized to more extensive periods of lockdown; (ii) compared to other investigations where pre-planning was possible, the number of our participants is relatively small. Nevertheless, under these very special circumstances, we were gratified to be able to include a relatively large number of athletes performing at a high level; (iii) our findings on the impact of the German strategy for containment of Coronavirus SARS-CoV-2 cannot necessarily be extrapolated to the situation in other countries; and, finally, (iv) our present findings are not necessarily valid for German athletes engaged in other types of sports (e.g., indoor and/or team sports), which may have experienced substantially different infrastructural and organizational consequences.

Furthermore, even though monitoring free movement by the Polar M430 has been shown to be reliable and may be suitable for characterizing changes in physical activity with time (Henriksen et al., 2019), accelerometers worn on the wrist might exaggerate or underestimate the level of activity. As also mentioned above, comparison of the algorithm utilized to assess sleep time with polysomnography in adolescents revealed that the former underestimates slightly but less so than many accelerometers designed for research purposes (Pesonen and Kuula, 2018).

Here, we were fortunate to be able to monitor important aspects of training characteristics, other physical activity, and sleep duration and with a smartwatch. Although other, more sophisticated methodologies can be utilized to assess these components of daily living in greater detail, the rapid development of the Covid-19 pandemic did not allow us to employ other approaches. Nonetheless, our current observations provide interesting insights into the effects of the lockdown on the training, other habitual physical activity, and the time spent sleeping by a group of highly trained German athletes.

## Conclusions

The data documented here reveal that following the German lockdown for containment of the Coronavirus SARS-CoV-2, highly trained kayakers and canoeists spent less overall time training each week (−27.6%) with, on average, shorter training sessions (−15.1%) and less light-to-moderate physical activity outside of training. Moreover, they performed more strength training sessions per week and all engaged in more training at intensities >82 and <60% of HR_peak_ while spending longer periods lying down and sleeping during the lockdown.

## Data Availability Statement

The raw data supporting the conclusions of this article will be made available by the authors without undue reservation.

## Ethics Statement

The studies involving human participants were reviewed and approved by the Department of Sport Science, University of Würzburg. Written informed consent to participate in this study was provided by the participants' legal guardian/next of kin.

## Author Contributions

All authors listed have made a substantial, direct and intellectual contribution to the work, and have approved it for publication.

## Conflict of Interest

The authors declare that the research was conducted in the absence of any commercial or financial relationships that could be construed as a potential conflict of interest.
